# Filling schemes at submicron scale: Development of submicron sized plasmonic colour filters

**DOI:** 10.1038/srep06435

**Published:** 2014-09-22

**Authors:** Ranjith Rajasekharan, Eugeniu Balaur, Alexander Minovich, Sean Collins, Timothy D. James, Amir Djalalian-Assl, Kumaravelu Ganesan, Snjezana Tomljenovic-Hanic, Sasikaran Kandasamy, Efstratios Skafidas, Dragomir N. Neshev, Paul Mulvaney, Ann Roberts, Steven Prawer

**Affiliations:** 1 School of Physics, The University of Melbourne, VIC 3010, Australia; 2 Melbourne Centre for Nanofabrication, Australian National Fabrication Facility, Clayton, VIC, 3168, Australia; 3 Nonlinear Physics Centre, Research School of Physics and Engineering, Australian National University, Canberra ACT 0200, Australia; 4 School of Chemistry and Bio21 Institute, University of Melbourne Parkville, VIC 3010, Australia; 5 Department of Electrical and Electronic Engineering, The University of Melbourne, Melbourne, VIC. 3010, Australia

## Abstract

The pixel size imposes a fundamental limit on the amount of information that can be displayed or recorded on a sensor. Thus, there is strong motivation to reduce the pixel size down to the nanometre scale. Nanometre colour pixels cannot be fabricated by simply downscaling current pixels due to colour cross talk and diffraction caused by dyes or pigments used as colour filters. Colour filters based on plasmonic effects can overcome these difficulties. Although different plasmonic colour filters have been demonstrated at the micron scale, there have been no attempts so far to reduce the filter size to the submicron scale. Here, we present for the first time a submicron plasmonic colour filter design together with a new challenge - pixel boundary errors at the submicron scale. We present simple but powerful filling schemes to produce submicron colour filters, which are free from pixel boundary errors and colour cross- talk, are polarization independent and angle insensitive, and based on LCD compatible aluminium technology. These results lay the basis for the development of submicron pixels in displays, RGB-spatial light modulators, liquid crystal over silicon, Google glasses and pico-projectors.

Conventional colour filters are made of dyes or pigments and exploit their particular absorption properties to produce different colours in displays^1^,^2^,^3^. However, as the pixel size is reduced to a few microns, conventional colour filters start to suffer from colour cross talk^4^. Furthermore, RGB filters utilizing existing technology must be fabricated in several steps, which presents severe challenges when trying to accomplish submicron scale alignment^3^. Coupling light into submicron scale pixels is also extremely difficult due to diffraction. These issues call for new strategies for the fabrication of submicron sized colour pixels.

Colour filters based on plasmonic effects^5^,^6^,^7^,^8^,^9^,^10^,^11^ are superior to conventional filters, especially when the pixel size is reduced to a few microns in size. This is because dye based filters cannot be made much thinner than several hundred nanometres because of their low absorption coefficients and because fabrication of each of the three dye filters for RGB colour schemes demands complex lithographic processes^5^. A further advantage of plasmonic filters is that they are tunable across the entire visible spectrum using just a single, optically thick metal film. Plasmonic filters based on aluminium (Al) are highly desirable compared to gold and silver for submicron scale colour pixels because Al is inexpensive, compatible with existing LCD technology and has good adhesion to many substrates, obviating the need for deposition of an extra adhesion layer, thus simplifying fabrication^12^,^13^. There are other advantages to using Al such as its lower optical loss in the 400 to 500 nm spectral range, due to its high bulk plasma frequency^13^,^14^,^15^. In principle, plasmonic filters are recyclable, have reduced cross talk, are durable at high temperature and are resilient to prolonged exposure to ultraviolet radiation^5^,^14^,^15^. It is also possible to directly couple light into the structures when submicron scale plasmonic filters are used as colour pixels. Recently, micron-sized, hole-based, plasmonic filters of Al were shown to exhibit transmission coefficients of more than 35%^5^. A hole-array based plasmonic filter design is superior to that based on slits or lines as the former is polarization independent^15^,^16^,^17^,^18^,^19^,^20^,^22^.

However, the development of submicron scale plasmonic RGB colour filters in Al for display technology remains a major challenge because there are several stringent requirements that the submicron scale colour filters need to satisfy. They should be made of a single component film (for easy integration onto a display panel); they must be transparent in the visible region of the spectrum (must operate in transmission mode); they must be polarization independent (in order to maximize the transmission efficiency); they should also be angle insensitive (the filtered colour should be same for any angle of excitation); they should exhibit reduced colour crosstalk by avoiding multiple spectral transmission peaks and finally, each RGB pixel should have the same size at the submicron scale. Even if all the above criteria are met, the design of the submicron pixels for display applications is not possible unless pixel boundary errors are eliminated at the submicron scale.

Our study consists of five key steps: (i) finding the minimum plasmonic filter size required to define a submicron scale pixel using simple holes in aluminium; (ii) illustrating pixel boundary errors (or filter boundary errors) at the submicron scale; (iii) developing a filling scheme to eliminate the pixel boundary errors (iv) demonstrating a 430 nm × 392 nm plasmonic filter (v) developing an angle insensitive submicron scale filter of size 690 nm × 632 nm based on a coaxial hole-coaxial hole (CH-CH) filling combination by tuning surface plasmons and Fabry-Perot resonances to eliminate pixel boundary errors.

## Results

### Investigation of minimum number of holes required to define a submicron scale colour filter

**Figure 1:**
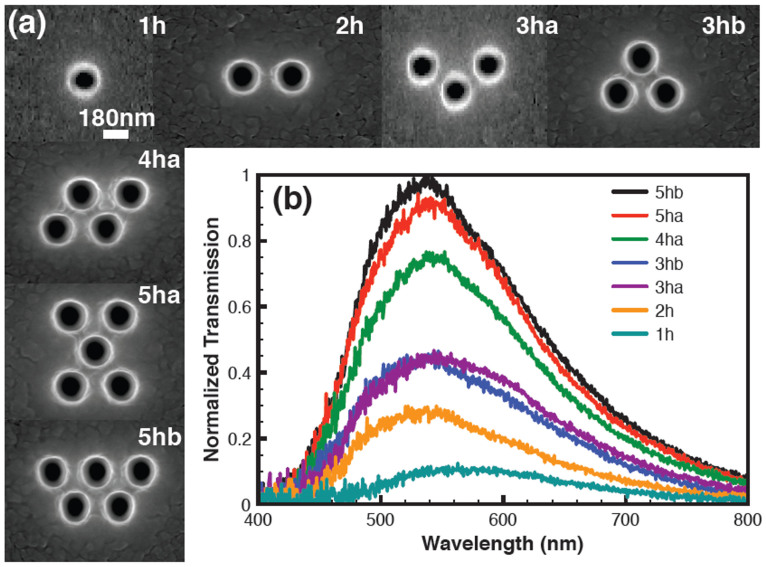
Investigation of the minimum number of holes required to define a submicron pixel (a) SEM images of a series of pixels composed of 1 to 5 holes in a hexagonal array (1h-5hb). Holes are 180 nm diameter with a 330 nm pitch fabricated in Al. (b) Experimental transmission spectra as a function of wavelength, showing the filtering effect as a function of the number of holes. An unpolarized white light source was used for the measurements.

We start by finding the minimum plasmonic filter size required to define a submicron scale pixel using simple holes in aluminium. A hexagonal arrangement is used for the submicron filter design rather than a square arrangement because the wavelength interval between the first two surface plasmon resonance peaks in a hexagonal array is larger than that in a square array^19^,^20^,^21^. For a hexagonal arrangement, the distance from the central hole to the next row of holes is the same. Since the surface plasmon resonance is sensitive to the pitch, contributions from all holes add constructively. For a square array, the pitch is different in the diagonal direction. The difference in pitch causes multiple peaks and a reduction in the overall transmission intensity (see Supplementary Information Section.1 and Fig. S1 for details). We have fabricated from one to five holes with different geometries in Al films, which are deposited on a glass wafer (fabrication details are given in Methods). The hole diameter is chosen to be 180 nm and the array pitch 330 nm in order to create a resonance peak close to 540 nm. Fig. 1b shows the variation in transmission peak intensity as a function of the number of holes and their geometry. These spectra have been collected from each of the single pixels shown in Figure 1. It is clear from the spectrum that colour filtering by a single-hole based pixel is broad and weak. Spectral filtering is already observed with two holes, as is evident from the slight increase in the transmission peak and the blue-shift induced by plasmon coupling. This is, however, not sufficient to produce a satisfactory pixel because there is strong polarization sensitivity due to the elongated shape of the pixel. For three holes, there are two arrangements possible. The first one is an L shaped arrangement of holes (*3ha* in Fig. 1a) while the second one is a triangular hole arrangement (*3hb* in Fig. 1a). The transmission increases for both arrangements. It is interesting to note that the full width at half maximum (FWHM) for *3ha* is slightly larger than for *3hb* (Fig. 1a). For *3ha*, an isosceles triangle arrangement is used while in *3hb*, an equilateral triangle arrangement is utilized. Three-hole pixels clearly exhibit improved filtering, with transmission increasing from 0.1 (a.u) (for a single hole) to 0.45 (triple hole) and this pattern also gives a peak position at the designed wavelength (530 nm) as shown in Fig. 1. There is another obvious advantage to the equilateral triangle arrangement (3hb): it is polarization independent due to its symmetry^16^.

For four holes, we have considered only one geometry (*4ha* in Fig. 1a), as other possible geometries are not useful for the pixel development due to their irregular morphologies and lower symmetry. For five holes, there are two likely candidates: a rectangular geometry (*5ha* in Fig. 1a) and an elongated geometry (*5hb* in Fig. 1a). The peak lies at 530 nm for both structures (the same as for the four hole pixel, but with slightly increased transmission). The elongated *5hb* pixel has a higher transmission than the *5ha*, which again highlights the importance of optimizing the hole format for the pixels. We conclude that, after taking into account the transmission, line-width, minimum size and polarization insensitiveness, the minimum number of holes required for realizing a submicron colour pixel is three in a triangular arrangement as shown in Fig. 1 (a), 3hb.

**Figure 2:**
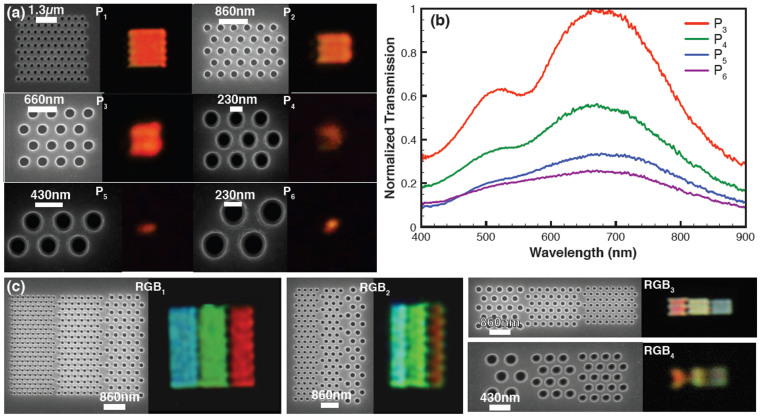
Development of submicron colour filters (a) SEM images of red colour filters of different sizes and the corresponding optical images (P_1_–P_6_) (100 × magnification). The filter size varies from 4.53 μm down to 660 nm. The optical images of the submicron scale plasmonic filters mimic the SEM images (pixel boundary errors) (P_1_–P_6_) (b) Experimental transmission spectra of the red submicron filters, P_3_ (1.73 μm × 1.53 μm), P_4_ (1.3 μm × 975 nm), P_5_ (1.3 μm × 602 nm), and P_6_ (660 nm × 602 nm) display a peak at 680 nm (c) SEM images of submicron colour filters with sub-RGB filters. The sub-RGB filter sizes for the red are 1.95 μm × 5 μm (RGB_1_), 875 nm × 5 μm (RGB_2_), 1.95 μm × 1.72 μm (RGB_3_) and 875 nm × 975 nm (RGB_4_). The corresponding optical images (100 × magnification) display pixel stitching errors at the boundaries (RGB_1_–RGB_4_).

Next, we have investigated whether there is any major shift in the peak wavelength position when the plasmonic filter size is reduced to submicron size. A red plasmonic filter with different sizes is fabricated (the size is varied from 4.53 μm to 660 nm) for the study (optimization of parameters is given in the Supplementary Information Section. 2 and Fig. S2). Figure 2a shows SEM images of these red filters, along with the corresponding images obtained under an optical microscope in transmission mode with 100 × magnification. The images and the related spectra confirm the successful operation of the red colour filter at a submicron scale. Fig. 2b displays the transmission spectra obtained for filters P_3_ (1.73 μm × 1.53 μm), P_4_ (1.3 μm × 975 nm), P_5_ (1.3 μm × 602 nm), and P_6_ (660 nm × 602 nm). Surprisingly, the peak position of the plasmons resonance remains around 680 nm for all the filter sizes. Here, the transmission at shorter wavelengths is dominated by photonic modes supported by the holes. The photonic modes are due to strongly localized field distributions inside the hole itself^5^ and is the reason for the occurrence of further minor peaks at shorter wavelength evident in Fig. 2b. A coaxial hole geometry instead of a hole geometry could suppress these photonic modes^5^. This could be realised by a structure composed of concentric, cylindrical holes. The resonance in such a geometry is dominated by Fabry-Perot resonances.

### Pixel boundary errors at the submicron scale

As demonstrated above, an equilateral triangular arrangement of holes defines the simplest, polarization-insensitive filter. Thus each plasmonic pixel should be triangular in geometry and is suitable for displays where triangular pixels are used. However, most of the current, state-of-the-art display technologies utilise rectangular or square pixels. Realization of a rectangular or square filter is challenging when a triangular (hexagonal) arrangement of holes is used. From Fig. 2, the optical image of the submicron scale plasmonic filters follows its own SEM image and this is termed a pixel boundary error. It is clear that there are irregularities at the edges of the submicron filters, due to the hexagonal arrangement of holes – this makes the design of a rectangular or square submicron scale filter an enormous challenge. The errors are visible even for a filter size of 4.53 μm as shown in Fig. 2a (P_1_). As the filter size decreases from 4.53 μm × 3.58 μm to 660 nm × 602 nm (P_1_–P_6_ in Fig. 2a) the filter boundary errors become more prominent. For example, the optical image of the *red* filter evinces a crescent shape when the size is reduced to 1.3 μm × 975 nm as shown in the Fig. 2a (P_4_).

We have further studied the combined effect of RGB filters when submicron scale RGB filters are placed together. The submicron scale filters with sub-RGB filters of different colours are fabricated (see Methods) based on computational studies using a finite number of holes (Supplementary Information Section. 2 and Fig. S2). Figure 2c shows SEM images of the fabricated filters. When the same number of holes is used for making each sub-RGB filter, the filter size is different for each red, green and blue filter. This is due to the different pitches required to generate the three different colours. In order to keep each sub-RGB pixel size almost the same, we have adjusted the number of holes comprising the red, green and blue filters, as shown in Fig. 2c. The sub-RGB filter sizes for the red are 1.95 μm × 5 μm (RGB_1_), 875 nm × 5 μm (RGB_2_), 1.95 μm × 1.72 μm (RGB_3_) and 875 nm × 975 nm (RGB_4_). Fig. 2c also shows the corresponding optical images (100 × Magnification). We have placed each filter with a separation of 40 nm. The optical images reveal irregularities with a zig-zag shape at the filter boundaries of the submicron RGB filter. The pixel boundary errors at the edge of each submicron scale colour filter lead to macroscale pixel stitching errors when the RGB filters are juxtaposed with nanometre spacings.

These pixel boundary errors and the subsequent stitching errors cause blurring, irregularities and lack of sharpness when images are exhibited on displays or are projected onto screens. For submicron pixel sizes, this effect is significant and is a major challenge for practical display development as shown in Fig. 2c. Notably, these errors are present irrespective of the spacing between each submicron scale colour filters and are also present even if the pixels are joined seamlessly.

### Elimination of the pixel boundary errors via a filling scheme

**Figure 3:**
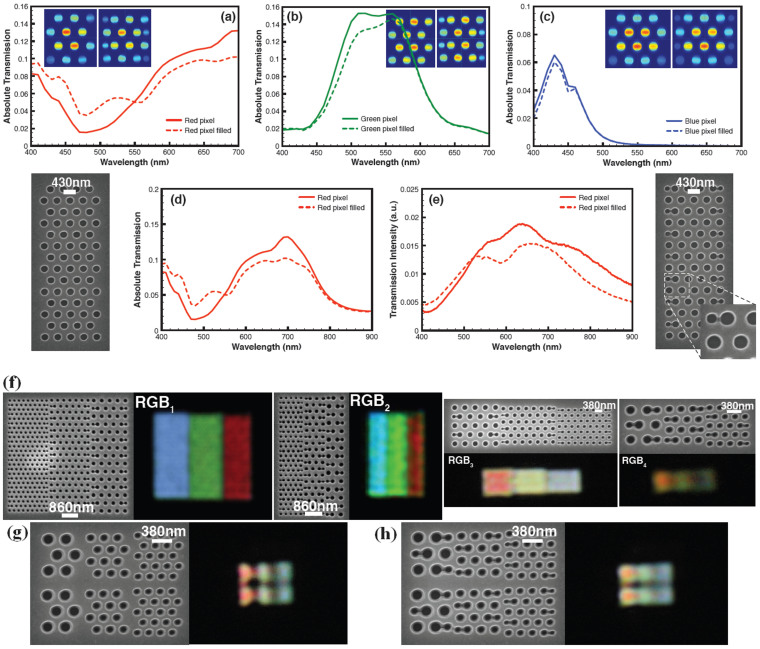
The pixel filling scheme. Numerically simulated transmission spectra before and after filling using 3 × 4 hole matrices for (a) a red filter (b) a green filter (c) a blue filter with wavelength swept from 400 nm to 700 nm (d) a red filter with wavelength swept from 400 nm to 900 nm before and after filling. The inset images in (a), (b) and (c) show the normalized electric field densities on the holes before and after filling (e) Experimentally measured transmission spectrum from a red pixel fabricated with and without filling and the corresponding SEM images on left and right (f) SEM images of submicron RGB colour filters of different sizes after the filling scheme and the corresponding optical images show boundary errors eliminated at the pixel boundaries without any colour cross talk. The sub RGB filter sizes for the red are 1.95 μm × 5 μm (RGB_1_), 875 nm × 5 μm (RGB_2_), 1.95 μm × 1.72 μm (RGB_3_) and 875 nm × 975 nm (RGB_4_) (g) SEM image of an array of the submicron scale filters with the red pixel size of 875 nm × 975 nm and the corresponding optical image shows three different colours red, green and blue (RGBs) without the filling scheme. The corresponding optical image shows the crescent shape of the red filter (the optical image follows its own SEM image geometry) and the boundary errors at the boundaries of each filter/pixel (h) SEM image of the same submicron scale filters after the filling scheme and the corresponding optical image showing the errors are eliminated (×100 magnification).

To circumvent the stitching issues for sub-micron pixels, we introduce a novel filling scheme. The filling scheme eliminates the errors by introducing extra holes into the colour filters as shown in Figs. 3a, 3b and 3c. The vacant spaces in the filter which cause the zig-zag irregularities at the boundaries are filled with holes with a suitable hole diameter and pitch; these produce a minimal effect on the resonance wavelength, and no colour deterioration. Finite element calculations were used to optimize the hole diameter and pitch for each pixel structure. The first step in the optimization is choosing a pitch for the inserted holes. The pitch is selected in such a way that the filter attains a more regular shape at the filter boundaries and such that the selected pitch does not introduce any new peaks or shifts in the resonance. Then the hole diameter used for the filling is varied to obtain the minimum boundary error. As the diameter of the added holes increases, minor peaks appear and also small shifts in the peak resonance occur. For the red filters, the boundary error is eliminated using a hole diameter of 170 nm, placed at a distance 60 nm away (edge to edge) from the last hole in the horizontal direction and then vertically repeated (Fig. 3a, left inset). In this way, the inserted holes and existing holes have a 372 nm pitch in the vertical direction and a 260 nm pitch in the horizontal direction. For the red, green and blue colour filters, 3 × 4 holes (filter size – 1346 nm × 1324 nm for red, 1005 nm × 1037 nm for green and 770 nm × 795 nm for blue) are used.

We have simulated the transmission spectrum for the red filter using the 3 × 4 hole structures (Fig. 3a) to make sure that there is no additional resonance (no colour cross-talk) created by the insertion of the filling holes. The same method has been followed for both the green and blue filters. For the green filter, the hole diameter used for filling is 90 nm with 285 nm and 195 nm pitch in the vertical and horizontal directions respectively (Fig. 3b right inset). The blue filter is filled with 60 nm holes with pitch 225 nm vertically and 160 nm horizontally (Fig. 3c right inset). In the case of the red filter, the 260 nm pitch causes a small rise in transmission in the blue-green region as shown in Fig. 3a. For the green filter, the 195 nm pitch again produces a small peak towards the blue region of the spectrum. However this peak is outside the filter operating wavelength range 400 nm to 700 nm (Fig. 3b). For the blue filter, there is no extra peak created in the visible spectrum by the filling scheme (Fig. 3c). The transmission reduces by 26% (red), 7% (green) and 6% (blue) of the peak (Fig. 3a–c) due to the presence of the defect, although the total number of holes is increased (Fig. 3a–d). The filling effect under the optical microscope (50 × magnification) is more noticeable for the red filter than for the green or blue filters. This is because, for the red filters, the hole diameter and pitch used are bigger than those employed for the green and blue filters. However, small boundary errors are still present for the green and the blue pixels. For the red filter, the simulated transmission spectrum has been computed from the wavelength range 420 nm to 900 nm before and after filling (Fig. 3d) which confirms that there is no extra peak introduced by the filling scheme except for a weak feature in the blue region. Based on these simulation results, we have also fabricated a red filter with and without filling. Figure 3 shows the SEM image of the red filter before filling (left side of Fig. 3d) and after filling (right side of Fig. 3e). The inset shows the magnified SEM image to highlight that there is no overlapping of holes. Figure 3e shows the transmission spectrum of the fabricated red filter. This result matches the simulated spectrum for the red filter (Fig. 3d) except for a small peak very close to 400 nm. This peak does not appear in the experimental plot because our spectrometer and light source used are not sensitive enough below 400 nm (U.V region). Since the pixel operates in the visible region, this peak will not cause any degradation in the colour. This peak can be pushed away from the visible region (400 nm to 700 nm) by suitable optimization of the hole diameter and the pitch.

Based on the above simulations and experimental results, we have fabricated submicron RGB colour filters with no pixel boundary errors. Figure 3f shows the SEM images and the corresponding optical images of the RGB colour filters. The images in Fig. 3f clearly show that there are no pixel boundary errors in comparison to Fig. 2c and also prove that the removal of such undesirable boundary effects is achieved with no colour cross talk. Furthermore we have fabricated a small array of the submicron filters to show the impact of the filling scheme. Fig. 3g shows an array of sub RGB filters without the filling scheme, where the optical image of the red filter size is 875 nm × 975 nm. The optical image of the red submicron scale filter shows a crescent shape (the same as its own SEM image in Fig. 3g), and the boundary errors are visible for the green and blue submicron scale filters. The filling scheme can eliminate the errors as shown in Fig. 3h, where the crescent shape is converted to a rectangular shape. However, the colours observed at the submicron scale do not appear as bright as colours at the micron scale.

### The filling scheme on a 430 nm × 392 nm size colour filter

**Figure 4:**
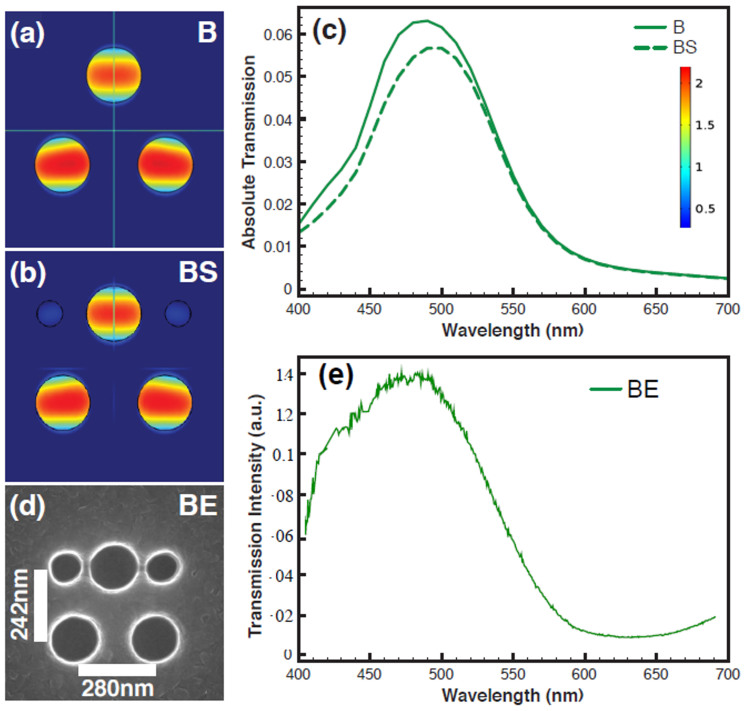
A submicron scale pixel with size 430 nm × 392 nm with and without filling scheme (a) The simulated submicron scale colour filter with size 430 nm × 392 nm in Al using an equilateral triangular geometry (hole diameter 150 nm, pitch 280 nm and Al film thickness 130 nm). The figure shows normalized electric field (E-field) densities on holes (b) The same filter after filling scheme using 70 nm diameter holes (c) Simulated transmission spectra from the same submicron scale colour filter with and without filling (d) SEM image of the fabricated submicron scale colour filter based on the simulation results with size 430 nm × 392 nm in Al using a triangular fashion after filling (e) Experimentally obtained transmission spectra from the fabricated 430 nm × 392 nm submicron scale filter.

Based on results obtained from the above filling scheme, we have shown that the same filling scheme can be applied to a filter size of 430 nm × 392 nm. The filter is designed using three holes with a hole diameter 150 nm and pitch 280 nm in a triangular fashion to obtain a resonance at 490 nm. Fig. 4a and 4b show the simulated 430 nm × 392 nm colour filter with and without the filling. For the filling, the hole diameter used is 70 nm and pitch 195 nm as shown in the Fig. 4b. Fig. 4c shows the simulated transmission spectra from the 430 nm × 392 nm colour filter with and without filling. The spectra show that even at the submicron scale, the optimized filling scheme does not introduce any prominent peaks or cause any colour cross talk or prominent shift in the peak wavelength. We have shown in Figs S3 and S4 of the Supplementary Information that study of the polarization insensitivity after the introduction of the filling scheme at submicron scale. Based on the simulation results, we have fabricated a submicron scale filter with size 430 nm × 392 nm employing the filling scheme. Fig. 4d and 4e show the SEM image of the fabricated submicron scale colour filter with size 430 nm × 392 nm and the experimentally measured transmission spectrum, which shows the peak wavelength at 490 nm. From Fig. 4e, it is clear that the experimentally obtained spectrum is in agreement with the simulated spectrum, both give the peak wavelength at 490 nm without introducing any new major peak or colour cross talk even after taking into account the fabrication tolerances (the diameter of holes used for the filling is a few nanometres wider than 70 nm). The same scheme can be applied to both the red and green submicron filters. The diameter should be properly optimized for the filling scheme at the submicron scale as a very small diameter hole will not completely eliminate the boundary error while very big diameter holes can introduce a small shift or minor extra peak in the resonance. The results show that it is possible to overcome boundary errors at the submicron scale without colour cross talk using the filling scheme.

## Discussion

We have used a simple, hole-based geometry to illustrate the importance of pixel boundary effects and stitching errors and we have also presented a filling scheme for correcting this effect. However, the hole-based, submicron-scale filter design is angle dependent i.e., the transmission wavelength depends on the angle of incidence of light impinging on the filter. If the hole array were used in a display, the filter would transmit the designed colour at normal incidence while different colours would be filtered at oblique incidence. Figure S5a in the Supplementary Information shows the simulated shift in the resonance peak with respect to the angle of incidence for a hole based green filter. Also, for the hole based design, different colours are obtained by varying mainly the pitch and to a lesser extent the hole diameter. This variation in the pitch necessitates different sizes for each RGB filter when a submicron filter is designed using a three hole geometry as shown in the Figs. 3f and 3g. Due to the fact that the surface plasmon resonance depends on the pitch, the extra holes used for filling do cause a small reduction in the transmission of the filter, as shown in Fig. 4c.

To overcome all the above issues, and satisfy all the requirements for submicron scale filter development, we have developed coaxial hole (CH) based submicron filters in aluminium by combining and tuning surface plasmon resonance with Fabry–Perot resonances, which meet all the desired properties including angle insensitivity.

In a CH plasmonic filter, the transmission peaks are caused by Fabry-Perot resonances and planar surface plasmons (PSPs)^23^,^24^. However, the resonance peak wavelength is determined largely by the Fabry-Perot resonance condition^23^,^24^,^25^,^26^,^27^,^28^. The Fabry-Perot resonance is dependent on the geometry of the holes and thickness of the film, while the surface plasmon resonance is dominated by the pitch of the two dimensional gratings. In the case of a CH filter, the transmission peaks are dominated by Fabry-Perot resonances in the cylindrical cavity (cylindrical surface plasmons) of the metal film with finite thickness and two end faces. The desired transmission peak can be estimated using the equation *l* = (nπ − θ)/β^27^, where *l* is the thickness of the metal film, *n* is the order of the Fabry-Pérot resonance, θ is the phase of reflection constant and *β* is the propagation constant. By keeping the metal thickness (*l*) constant, the propagation constant (*β*) can be varied with different CH aperture sizes to tune the transmission peak. Since the Fabry-Perot resonances are associated with localized surface plasmons^29^, there is no shift in the resonance peak with respect to the angle of incidence for the CH based filters (Fig. S5b in the Supplementary information). In the CH based filter design, tuning of the resonance peaks (colours) is achieved by varying the inner (R_1_) and outer radius (R_2_) of the coaxial hole while keeping the pitch the same. In addition to all of these advantages of CH based filters, the boundary error can be completely eliminated with no colour cross talk and transmission intensity reduction. This is because the filling doesn't affect the resonance much due to its pitch because the resonance is dominated by the Fabry-Perot effect, which is independent of pitch.

### Submicron scale colour filters using CH-CH filling combination

**Figure 5:**
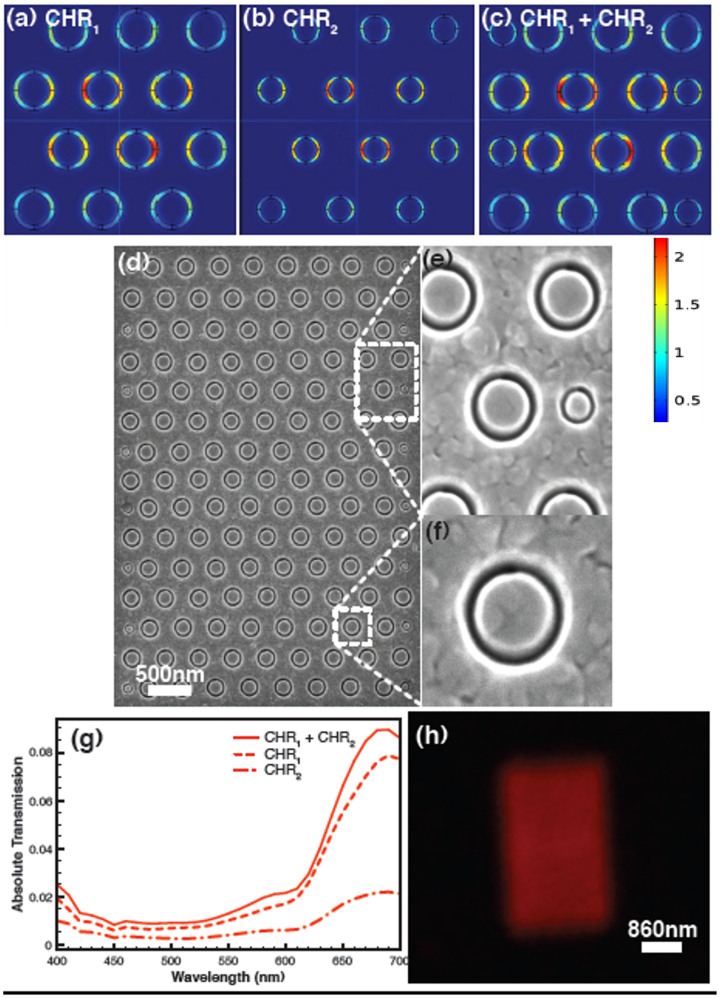
Filling scheme for the coaxial hole (CH) based filter (a) Simulated red colour filter CHR_1_ shows normalized electric field (E-field) with the boundary error (b) Simulated submicron red colour filter CHR_2_ shows normalized electric field with the boundary error (c) Combination of CHR_2_ and CHR_1_, ie CHR_2_ + CHR_1_ showing normalized electric field, where the boundary error is fully eliminated (d) SEM image of the fabricated red colour filter CHR_2_ + CHR_1_ (e) Magnified SEM image of the CHR_2_ + CHR_1_ showing CHR_1_ and CHR_2_ (f) Magnified SEM image of one CH (g) Computationally obtained transmission spectra from CHR_1_, CHR_2_ and CHR_2_ + CHR_1_ combination (h) Optical image of the filter CHR_2_ + CHR_1_ combination shows no pixel boundary error.

A red colour filter is obtained by choosing R_1_ = 130 nm and R_2_ = 100 nm (CHR_1_) to obtain a peak wavelength at 690 nm (Fig. 5a). The pitch is chosen to be 430 nm because this pitch also corresponds to resonance towards the red region of the visible spectrum. So a red submicron filter is designed (CHR_1_), which meets all the criteria including the angle insensitivity, except the filter boundary error as shown in Fig. 5a. Here, to eliminate the filter boundary error, another CH geometry is designed with R_1_ = 90 nm and R_2_ = 70 nm (CHR_2_), which is smaller than the CHR_1_ as shown in Fig. 5b. Both the CHR_1_ and CHR_2_ designs yield peak wavelengths of 690 nm. Then the CHR_2_ is used for filling the CHR_1_ filter to create a new combination filter, which we denote ‘CHR_2_ + CHR_1_’, as shown in Fig. 5c. Figure 5g shows the computationally obtained peak with only CHR_1_, CHR_2_ and with the CHR_2_ + CHR_1_ combination.

The filter boundary error is fully eliminated with no major additional peaks and also with enhancement in the transmission intensity. The CH-CH based filling scheme is ideal because CHR_2_ used for filling doesn't affect the resonance much due to its pitch because the resonance is dominated by the Fabry-Perot effect, which is independent of pitch. To demonstrate this experimentally, we have fabricated a red colour filter based on the CH combination. Figure 5d shows the SEM image of the CHR_2_ + CHR_1_ combination filter. Figures 5e and 5f show the magnified SEM image of the filter. Figure 5h shows an optical image of the CHR_1_ + CHR_2_ combination filter showing no filter stitching error and colour cross talk at the boundaries. The same method can be repeated for the green and blue colour filters.

### The CH-CH filling combination on 690 nm × 632 nm size filter

**Figure 6:**
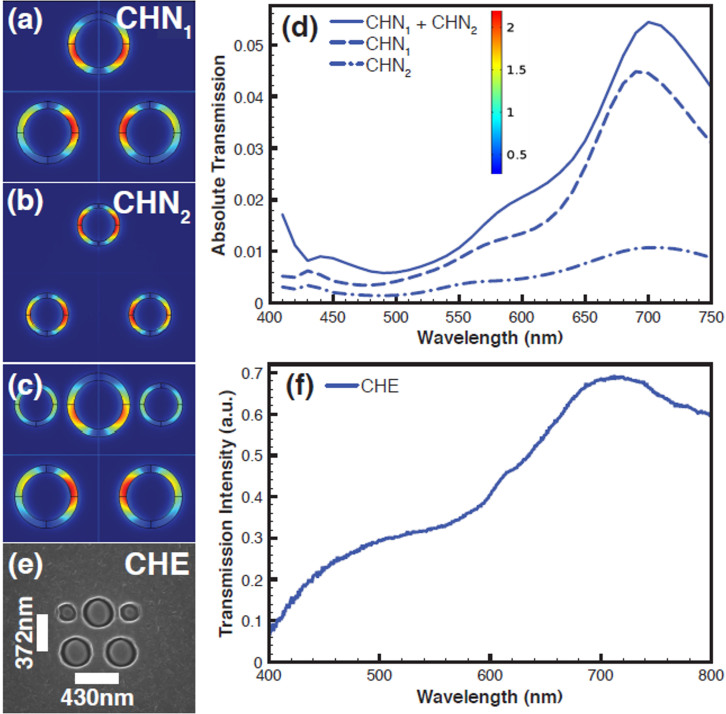
A CH-CH combination based submicron scale filter with size 690 nm × 632 nm (a) The simulated CH submicron scale colour filter with size 690 nm × 632 nm in an equilateral triangular geometry using three CHs with the inner and outer radii of 130 nm and 100 nm respectively and the pitch of 430 nm (CHN_1_) (the figure shows normalized electric field (E-field) on the CHs) (b) CHN_2_ with the inner and outer radii of 90 nm and 70 nm (c) The filter after the CH-CH combination scheme (d) Simulated transmission spectra from the CH submicron scale colour filters (CHN_1_, CHN_2_ and CHN_1_ + CHN_2_ combination (e) SEM image of the fabricated CH based submicron scale colour filter CHE with size 690 nm × 632 nm in Al using CHN_1_-CHN_2_ combination (f) Experimentally obtained transmission spectrum from the CHE with size 690 nm × 632 nm.

As a next step, we show the CH-CH based filling scheme applied to a submicron filter using the equilateral triangular geometry with a size 690 nm × 632 nm (Fig. 6). As shown in Fig. 6a, the inner and outer radii are tuned to 130 nm and 100 nm respectively and the pitch to 430 nm in order to obtain a resonance in the red region at 690 nm (CHN_1_). For filling, as mentioned in the above section a small CH with outer and inner radii 90 nm and 70 nm is used as shown in Fig. 6b (CHN_2_). Then CHN_1_ and CHN_2_ are combined to create the submicron scale filter CHN_1_ + CHN_2_ as shown in the Fig. 6c. Fig. 6d shows the computationally obtained spectra from the submicron scale filter with and without the CH-CH filling scheme. The spectra show that the CH-CH filling scheme does not introduce any new peaks, nor does it cause any colour cross talk, even for a filter size of 690 nm × 632 nm.

The transmission intensity increases after the filling scheme is implemented as shown in Fig. 6d. We have shown polarization insensitivity study of the CH triangular geometry at the submicron scale in S6 of the Supplementary information. Based on the simulation results, we have fabricated a submicron scale filter with the size 690 nm × 632 nm using the CH-CH filling scheme. The SEM image of the filter is shown in Fig. 6e (CHE). Fig. 6f shows the experimentally measured transmission spectrum from the 690 nm × 632 nm size filter, which exhibits a peak at 690 nm and this matches the simulation results, except for the presence of two minor peaks at 400 nm and 570 nm. These peaks can be suppressed further by tuning the pitch and the CH geometry. Using the CH-CH filling scheme, each submicron scale RBG filter can be made of the same size because the colour (the resonance) is tuned mainly by varying the gap between outer and inner radii keeping the pitch same. These results show that the CH-CH filling scheme is the best strategy to develop submicron colour filters with uniform size for each RGB filter without stitching errors, which are also polarization independent, angle insensitive and based on LCD compatible aluminium technology. For large area fabrication, electron beam lithography or nanoimprint lithography can be used, so that the filling scheme can be easily integrated into the design.

In summary, we have presented a pathway for the development of submicron plasmonic colour filters. We initially determined the minimum plasmonic filter size required to define a submicron scale pixel using simple holes in aluminium. The results showed that the minimum number of holes required for realizing a polarization independent submicron colour filter is three in an equilateral triangular arrangement. This enables development of triangular pixels in displays. But the majority of state-of-the-art displays use rectangular or square shape pixels. We then demonstrated that development of rectangular or square shape pixels is hindered by boundary errors and stitching errors at the submicron scale. These errors cause blur, reduced sharpness and discontinuities in the displayed images. In order to eliminate these errors, we have developed a simple but powerful filling strategy by combining surface plasmon and Fabry-Perot resonances. Using both computational methods and experiments, a submicron scale colour filter of 430 nm × 392 nm size was fabricated without any colour cross talk or pixel boundary errors. We then discussed drawbacks of hole based designs as they are angle sensitive with transmission wavelength depending on the angle of incidence. Furthermore, it is difficult to obtain the same filter size for each RGB filter at the submicron scale because the pitch is varied in order to generate different colours, and the filling scheme reduces the transmission intensity. In order to address all these issues, we developed the CH-CH filling combination scheme and demonstrated a submicron scale filter with the size 690 nm × 632 nm, which completely eliminated the boundary and stitching errors with increased transmission without any colour cross talk. In addition, these filters are polarization independent, angle insensitive and based on LCD compatible aluminium technology. These submicron colour filters may have applications in displays, projectors, spatial light modulators (SLM), liquid crystal over silicon technology (LCOS) and wavefront sensors.

## Methods

### Focused ion beam (FIB)

A glass wafer of thickness 500 μm was used for fabricating the submicron scale plasmonic filters for different filter geometries. The glass substrate was cleaned using acetone, isopropyl alcohol and deionized water. A 150 nm Al film was evaporated at the rate of 0.5 Å/s on the wafer using Intlvac Nanochrome II electron beam evaporation system. Both the coaxial holes (CHs) and the holes were milled into the Al using an FEI Helios NanoLab 600 Dual Beam focused ion beam (FIB). For holes, the current was varied from 1.5 pA to 28 pA depending on the feature size by setting the voltage at 30 kV. The sample was loaded immediately from the evaporator to the FIB to avoid the Al being oxidized. After the milling the sample was immediately coated with 50 nm SiO_2_ using plasma enhanced chemical vapor deposition (PECVD) to prevent the Al from oxidizing and for matching the index with the glass substrate to enhance transmission. We have fabricated the same feature at different location of the sample and also in different samples. We have observed the same colour from all these features. From this, we understand that the a few submicron roughnesse will not shift the resonance to change any colour. For the coaxial hole (CH) array, the Al was evaporated at the rate of 0.4 Å/s followed by milling using the FIB. The current used for the milling was 1.5 pA and the voltage 30 kV. The resonance peak of the CH array was very sensitive to any dielectric film on the top. The dielectric sensitivity of the resonance peak for the CH filters can be used for further tuning the peak wavelength position by depositing materials such as MgF_2_ (refractive index ~ 1.38) or SiO_2_(refractive index ~ 1.5). We have taken cross section of the fabricated arrays after the milling to make sure that 150 nm Al was milled through. We have done this after depositing platinum on the structure to protect the structure while taking a cross section and getting better contrast between the glass and Al. We have also used 130 nm thick Al film for making submicron filters.

### Optical characterization

The white excitation light from a halogen lamp (100 W) was focused onto the sample using a Nikon TE2000-S Eclipse inverted microscope. The transmitted light through the sample was collected via a 40 × dry objective lens with an iris diaphragm (numerical aperture 0.6) and then focused onto the entrance slit of a MicroSpec 2150i imaging spectrometer coupled to a thermoelectrically cooled (−60°C) CCD (charge-coupled device) (Acton Pixis 1024B Exelon). To get spectra from small feature sizes (<1 micron) a fiber based spectrometer to measure the transmission percentage from submicron features. We have used collimated illumination and different NAs (~0.3–0.7) depending on the feature size. The spectrum for the coaxial geometry was same for all NAs, which is due to the angle insensitiveness of the geometry. The low image brightness of the features at submicron scale is due to the fact that they are beyond the resolution of an optical microscope. For the regular holes, transmission in the order 7–35% was observed (35% for large red colour filters ~ 8 μm and close to 7–10% for submicron red colour filters). For the coaxial holes, transmission varied between 5–62%, again the transmission reduced to 5–8% for submicron filters.

### Numerical simulations

The submicron scale plasmonic filters were computationally investigated using the finite element method (FEM) implemented in COMSOL MULTIPHYSICS. For hole based colour filters, the pitch and hole diameter was varied to get different colours. The simulation model to find the peak wavelength of the colour filters consists of 150 nm or 130 nm Al on a semi-infinite thick glass substrate. The Al was then covered with 50 nm silica (SiO_2_) and then semi-infinite air above the silica. We have used a finite number of plasmonic elements (simple holes or coaxial holes) in the Al and perfectly matched layer (PML) was used on four side walls. The light excitation was done from the substrate side. We have used S- parameters to find the transmission percentage (S_21_). The same model was used for CH based colour filters. For the CH based filters, the simulations were carried out with SiO_2_ and without SiO_2_ layer on the top of the Al. There was a considerable red shift in the peak with SiO_2_ for the CH based filters. The filters can also be fabricated without a SiO_2_ layer as a cap layer on the top of the Al. Then the optimization of parameters should be done using the effective refractive as mentioned in the Ref. [13] to take into account the oxide layer (Al_2_O_3_). A submicron colour filter with sizes 430 nm × 392 nm (for holes based design) and 690 nm × 632 nm (for the CH-CH based design) were designed using 3 holes and CHs in a triangular geometry.

## Author Contributions

R.R. conceived and implemented the idea and directed the project. R.R. prepared the sample. K.G. and S.K. assisted the sample preparation. R.R. and E.B. fabricated the device using FIB. S.C., A.M., R.R., D.N. and A.D.A. designed the experiments. A.M., S.C., R.R. and A.D.A. carried out spectral measurements. R.R. carried out simulations. A.R., A.D.A., S.T.-H. and T.J. contributed to simulations. R.R. and T.J. prepared the graphics. R.R., K.G. and A.D.A. carried out microscope experiments. R.R. wrote the paper and A.R., P.M., D.N., E.S., S.T.-H. and S.P. participated in the analysis of results, discussing and writing the manuscript. A.R., E.S., P.M., D.N. and S.P. supervised the research. All authors reviewed the manuscript.

## Supplementary Material

Supplementary Information Supplementary Information
