# Supplementary material for: Filling schemes at submicron scale: Development of submicron sized plasmonic colour filters
Source: Sci Rep. 2014 Sep 22;4:6435. doi: 10.1038/srep06435 (PMC4170198; doi:10.1038/srep06435)
Supplement: Supplementary Information [file srep06435-s1.doc]

**Supplementary Information**

**Filling schemes at submicron scale: Development of submicron sized plasmonic colour filters**

Ranjith Rajasekharan^1^, Eugeniu Balaur^2^, Alexander Minovich^3^, Sean Collins^4^, Timothy James^12^, Amir Djalalian-Assl^1^, Kumaravelu Ganesan^1^, Snjezana Tomljenovic-Hanic^1^, Sasikaran Kandasamy^2^, Efstratios Skaﬁdas^5^, Dragomir N. Neshev^3^, Paul Mulvaney^4^, Ann Roberts^1^ and Steven Prawer^1^

^1^School of Physics, The University of Melbourne, Victoria 3010, Australia

^2^Melbourne Centre for Nanofabrication, Australian National Fabrication Facility, Clayton,

Victoria, 3168, Australia

^3^Nonlinear Physics Centre, Research School of Physics and Engineering, Australian National University, Canberra ACT 0200, Australia

^4^School of Chemistry and Bio21 Institute, University of Melbourne Parkville, VIC 3010, Australia

^5^Department of Electrical and Electronic Engineering, The University of Melbourne, Melbourne, VIC. 3010, Australia

1. **Advantages of a hexagonal arrangement over a square arrangement at nanoscale**

To design nanometer plasmonic colour filters, we consider a hexagonal arrangement of holes in Al for the nanometer RGB colour filter design. A hexagonal (triangular) arrangement was selected over a square array because the wavelength interval between the first two surface plasmon resonance peaks in a hexagonal array is larger than that of a square array. This enables to select the desired transmission wavelength (desired colour) with no colour cross-talk by pushing any undesired peak away from the visible region (400 nm - 700nm). The spectral line width for the square arrangement is larger than the hexagonal arrangement, which causes increased colour cross talk for the square arrangent compared to the hexagonal arrangement. The transmission percentage is higher for the hexagonal arrangement compared to the equivalent the square arrangement and this effect is crucial at nanoscale. The hexagonal arrangement also has a higher fill factor compared to the square array for the same period and hence increased transmission efficiency. This fill factor is very important for the development of the nanometer filters where vacant spaces will cause performance degradation and reduced efficiency. The square array is extremely sensitive to fabrication tolerances. For example if the period in *x* direction is slightly different from *y* direction, the geometry is polarization sensitive. But, the hexagonal arrangement is resilient to fabrication tolerances.


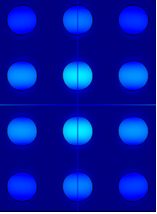

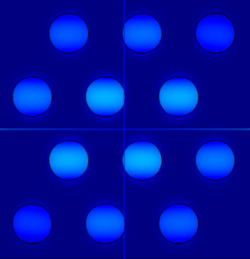


**Figure S1: Comparison of transmission spectra from a hexagonal arrangement and square arrangement for a green colour filter with 3× 4 holes**. The inset shows the square and hexagonal arrangement used for the simulation. The pitch was 330 nm for both the cases and kept number of holes same in both the arrangements. The simulation model consists of an Al film of thickness 150 nm on a semi-infinite glass substrate and the Al was covered with a 50 nm thick SiO_2_ layer. It is clear from the spectra that the hexagonal arrangement is far superior compared to the square arrangement in terms enhanced transmission and line width. The line width measured here was 150 nm for the square arrangement and 126 nm for the hexagonal arrangement. From these results, a hexagonal arrangement is essential for the nanoscale colour filter development.

1. **Optimization of parameters for plasmonic RGB filters**

We have used finite number of holes to find the required pitch and hole diameter for different colours. For the red, green and blue colour filters, 3× 4 holes (filter size – 1346 nm × 1324 nm for red, 1005 nm ×1037 nm for green and 770 nm × 795 nm for blue) were used for optimization using the finite element method implemented in COMSOL multiphysics software. The model consists of 150 nm thick aluminium (Al) on a semi-infinite thick glass substrate. Then the Al was covered with 50 nm silica (SiO_2_) and a semi-infinite layer of air above the silica. A perfectly matched layer (PML) was used around the substrate in four corners. A port was designed above and below the substrate with S parameters for finding the transmission spectrum and percentage of transmission. The illumination was from the top substrate side. Figure 2S shows optimized parameters for the filters using 3× 4 holes in a hexagonal fashion together with SEM image of the fabricated *INM* letters using the FIB.

**(a)**


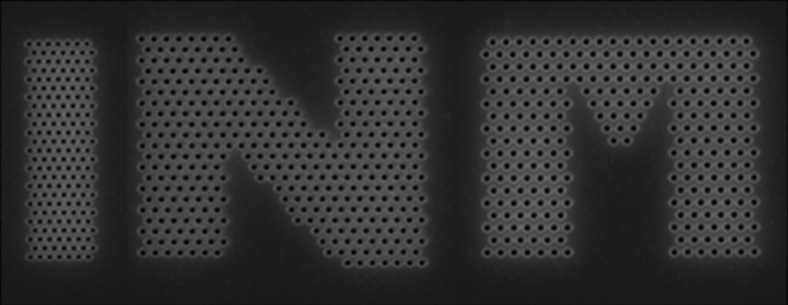

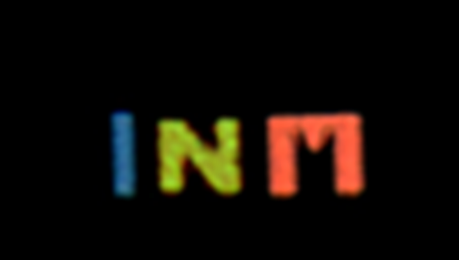


**(a)**

**(b)**

**(c)**

**860 nm**

**Figure S2: Optimisation of the parameters for red, green and blue colour filters using 3×4 holes** (a) Simulated transmission spectrum from 3 × 4 holes as a function of wavelength; red colour filter (pitch 430 nm and hole diameter 230 nm), green filter (pitch 330 nm and hole diameter 180 nm) and blue filter (pitch 260 nm and hole diameter 140 nm). The spectra show that three colours are distinguishable with minimal cross talk (b) SEM image of the fabricated *INM* letters using the FIB, based on the computationally obtained parameters. The size of the letter *I* is 1.82 µm × 5.53 µm. (c) The optical image of the INM under an optical microscope in transmission mode (× 20 Magnification) shows colour as per the simulation results.

**Figure S3: Nanoscale filter with size 430 nm × 392 nm under different polarizations**. (a) Transmission intensity versus wavelength for a nanoscale colour filter under three different polarization angles 0^o^, 45^o^ and 90 ^o^. The colour filter was designed using the three holes with the hole diameter 150 nm and pitch 280 nm in a triangular fashion to get a filter size of 430 nm × 392 nm. The resonance peak for the current design occurs at 490 nm. For the filling, the hole diameter used was 70 nm and the pitch 195 nm (centre to centre distance). The simulation model consists of an Al film of thickness 130 nm on a semi-infinite glass substrate, and the Al was covered with a 50 nm thick SiO_2_. From the Fig 3S it is clear that the variation in the peak of the resonance (peak of the transmission spectrum) is negligible for different polarizations after the filling scheme. But the polarization sensitivity increases as the hole diameter increases.

**Figure S4: Nanoscale filter with size 510 nm × 466 nm under different polarization for an un-optimized filling scheme** (a) Transmission intensity versus wavelength for a nanoscale colour filter under three different polarization angles 0^o^, 45^o^ and 90^o^. The colour filter was designed using the three holes with the hole diameter 180 nm and pitch 330 nm in a triangular fashion to get a filter size of 510 nm × 466 nm, and the resonance at 550 nm. For the filling, the hole diameter used was 90 nm and pitch 195 nm (centre to centre). These parameters were not optimised. The simulation model consists of an Al film of thickness 130 nm on a semi-infinite glass substrate and the Al was covered with a 50 nm thick SiO_2_. It is clear from the Fig 3S that even for an un-optimised filling scheme, the variation in the transmission spectrum is minimal for different polarizations after the filling scheme.


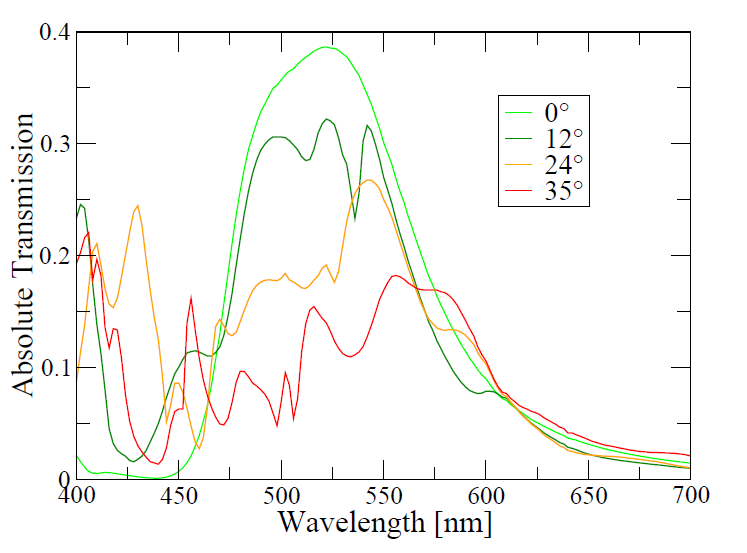

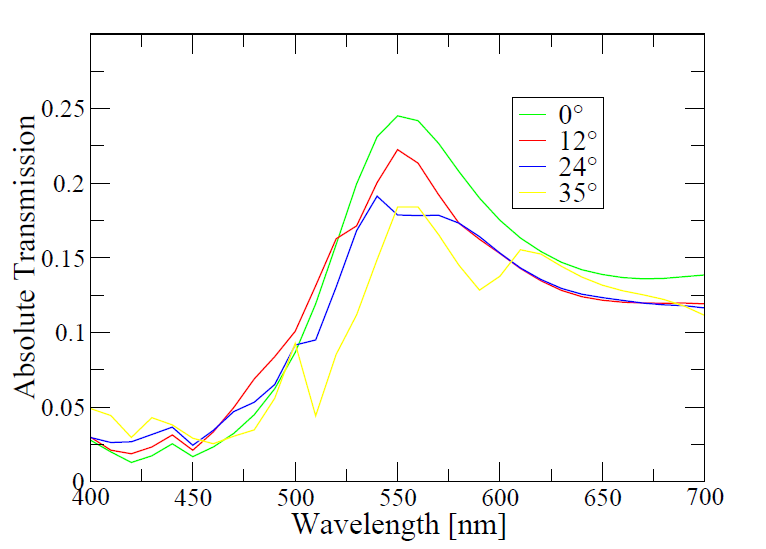


**(a)**

**(b)**

**Figure S5: Angle dependence of the nanometer filters developed using simple hole and cylindrical hole (CH) array** (a) For the simple hole array based filters, the peak wavelength shifts towards the red region as the angle of incidence increases. The angle was varied from 0 to 35 (0-60 degrees in full angle) (b) Angle insensitivity for the CH based filters. The resonance peak remains constant irrespective of the angle of incidence. The geometry consists of 150 nm thick Al on semi-infinite glass substrate. Then the Al was covered with 50 nm SiO_2_ on both the cases.

**Figure S6: A CH-CH combination based nanoscale colour filters with size 690 nm × 632 nm under three different polarizations** (a) Transmission intensity versus wavelength for a CH –CH combination based nanoscale colour filter under three different polarization angles 0^o^, 45^o^ and 90 ^o^. The colour filter was designed using the three CHs in a triangular fashion with inner and out radii of 130 nm and 100 nm respectively and the pitch to 430 nm to get the resonance in the red region at 690 nm (CHN_1_). For the filling, a small CH with outer and inner radii 90 nm and 70 nm is used (CHN_2_) with the same pitch. Then the CHN_1_ and the CHN_2_ are combined to get the nanoscale filter as shown in the inset with size 690 nm × 632 nm. The simulation model consists of an Al film of thickness 150 nm on a semi-infinite glass substrate and the Al was covered with a 50 nm thick SiO_2_. From the Fig 6S it is clear that the variation in the resonance (transmission spectrum) is negligible for different polarizations after the CH-CH filling combination scheme.
